# Ancestral stress programs sex-specific biological aging trajectories and non-communicable disease risk

**DOI:** 10.18632/aging.102848

**Published:** 2020-02-22

**Authors:** Mirela Ambeskovic, Yaroslav Ilnytskyy, Douglas Kiss, Cheryl Currie, Tony Montina, Igor Kovalchuk, Gerlinde A.S. Metz

**Affiliations:** 1 Canadian Centre for Behavioural Neuroscience, Department of Neuroscience, University of Lethbridge, Lethbridge T1K 3M4, Alberta, Canada; 2 Department of Biological Sciences, University of Lethbridge, Lethbridge T1K 3M4, Alberta, Canada; 3 Faculty of Health Sciences, University of Lethbridge, Lethbridge T1K 3M4, Alberta, Canada; 4 Department of Chemistry and Biochemistry, University of Lethbridge, Lethbridge T1K 3M4, Alberta, Canada

**Keywords:** sexual dimorphism, epigenetic regulation, prenatal stress, longevity, non-communicable disease

## Abstract

The incidence of non-communicable diseases (NCDs) is rising globally but their causes are generally not understood. Here we show that cumulative ancestral stress leads to premature aging and raises NCD risk in a rat population. This longitudinal study revealed that cumulative multigenerational prenatal stress (MPS) across four generations (F0-F3) raises age- and sex-dependent adverse health outcomes in F4 offspring. MPS accelerated biological aging processes and exacerbated sex-specific incidences of respiratory and kidney diseases, inflammatory processes and tumors. Unbiased deep sequencing of frontal cortex revealed that MPS altered expression of microRNAs and their target genes involved in synaptic plasticity, stress regulation, immune function and longevity. Multi-layer top-down deep learning metabolite enrichment analysis of urine markers revealed altered metabolic homeodynamics in MPS males. Thus, peripheral metabolic signatures may provide sensitive biomarkers of stress vulnerability and disease risk. Programming by MPS appears to be a significant determinant of lifetime mental health trajectories, physical wellbeing and vulnerability to NCDs through altered epigenetic regulation.

## INTRODUCTION

The world’s aging population is rapidly growing and by 2020 the number of individuals 60 years and older is expected to exceed the number of children and youth [1]. This sharp increase highlights the urgent need to identify strategies that support healthy aging. Approximately 88% of aged individuals in North America experience dramatic physical and mental health decline [2] which mainly results from accumulated cell and DNA damage acquired across the lifespan through interactions with adverse environmental and lifestyle conditions [2–5], raising chronic, non-communicable disease (NCD) risk. In fact, NCDs are the leading cause of death globally [6] and the main cause of premature morbidity and mortality [7].

Experiences in early life may lay the foundation for NCD suseptibility. The developmental origins of health and disease (DOHaD) hypothesis postulates that many common NCDs originate *in utero* by re-programming fetal physiological and metabolic responses with lifelong consequences on organ and tissue function [8]. Animal and clinical studies demonstrate that an adverse prenatal environment exacerbates hypothalamic-pituitary-adrenal (HPA) axis responsiveness; heightens risk for abnormal heart and kidney morphology and disease; increases blood pressure, cholesterol, insulin, and obesity; impairs mental health trajectories; and reduces lifespan [9–17].

The biological signatures linked to early life adversity are also transmitted across generations. Natural disaster and nutritional birth cohorts [18–21] as well as experimental studies [3, 22–29] have demonstrated that remote ancestral adverse experiences increase the risk of metabolic, cardiac and renal disease, and mental illness with a sex-specific bias. These adverse health outcomes are linked to epigenetic regulation, including altered microRNA (miRNA) expression [30–32]. Experimental studies revealed that multigenerational prenatal stress (MPS) in particular is more potent in programming the stress response than early life stress [15, 26], especially in male offspring. The recurrent gestational challenge in MPS models was shown to impair fine motor function, alter neuromorphology and induce hemispheric dominance shifts in males [3, 16], while improving fine function and promoting resilience in females [17]. Thus, MPS models may offer unique insights into evolutionary mechanisms of sex-dependent adaptation and resilience. Research has shown that ancestral biological memories of adverse experiences are linked to epigenetic modification, such as DNA methylation [30, 33, 34], histone modification [35–37] and miRNA expression [17, 26, 30–32]. Notably, the consequences of ancestral stress become particularly visible during early development and old age [16]. However, the impact of ancestral stress on physical and mental health during biological aging has not yet been demonstrated.

Here, we performed a controlled mixed longitudinal rat cohort study to examine the impact of recurrent stress reaching back across four generations (F0-F3) on lifetime health trajectories. We hypothesized that MPS in the F4 generation would lead to a behavioural phenotype of sex-specific stress vulnerability and resilience at young and old age. Moreover, we proposed particular vulnerability to NCDs in old age in association with up-stream epigenetic and down-stream metabolic biomarker signatures. The findings show that aging and MPS synergistically disturb the stress response and accelerate age-associated morbidity and mortality with sex-specific NCD incidence. By focusing on miRNAs as clinically valid and robust biomarkers [38–41] of aging, the present findings also address an urgent need to identify potentially pathogenic pathways underlying NCDs for early prediction and prevention in precision medicine approaches [42–45].

## RESULTS

### Physical and mental health outcomes and sensorimotor function

### MPS induced anxiety-like behaviours in young and aged males

**Figure 1:**
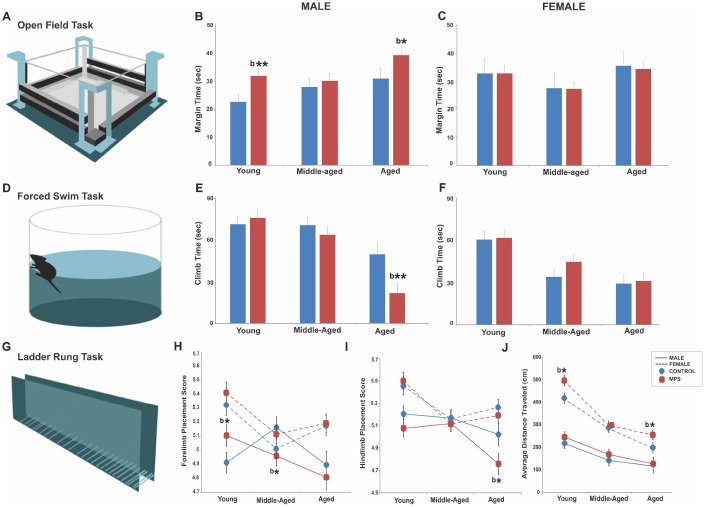
**MPS modifies the emotional and sensorimotor phenotype across the lifespan.** (**A**–**C**) MPS increased anxiety-like behaviours in young and aged males, but not in females. (**D**–**F**) MPS exacerbated the age-associated reduction in learned helplessness. (**G**–**I**) MPS induced sex- and age-specific effects in fore- and hindlimb skilled limb use. (**J**) Females showed higher locomotor activity than males at all ages. Asterisks indicate significances: *p<0.05. “b” indicates MPS effect. All data are presented as mean ± SEM.

MPS increased arousal and anxiety-like behaviour in males as a function of age. A mixed longitudinal assessment at 6 (young), 12 (middle-aged) and 18 months (aged) of age in the open field task showed increased anxiety-like behaviours with age in male rats only, especially in the MPS group. A three way-ANOVA showed main effects of AGE (F(2,116)=5.37, p<0.01), STRESS (F(2,116)=3.8, p<0.05) and SEX x STRESS interaction (F(2,116)=4.9, p<0.05). Aged animals spent more time in open field margins than the young (p<0.001) or middle-aged (p<0.05). MPS males spent more time in margins than CONTROLs at 6 months (p<0.01) and 18 months of age (p<0.05; Figure 1A, 1B). By contrast, margin time remained the same in females, with only a slight decrease in aged MPS females (p>0.05; Figure 1C). Thus, MPS altered life trajectories of anxiety-like behaviours in terms of the time spent in margins of the open field in males, with larger emotional changes early and later in life. By contrast, females were less severely affected.

### MPS induced sex-specific vulnerability to age-associated depressive-like behaviours

MPS reduced motivation as a symptom of depressive-like behaviours after midlife in males but spared middle-aged females. A mixed longitudinal assessment at 6 (young), 12 (middle-aged) and 18 months (aged) of age in the forced swim task showed an aging-associated increase in depressive-like behaviour as indicated by decreased climbing time in MPS aged males. A three way-ANOVA showed main effects of AGE (F(2,108)=29.8, p<0.001), SEX (F(2,108)=16.45, p<0.001), AGE x SEX interaction (F(2,108)=3.7, p<0.05) and SEX x STRESS interaction (F(2,108)=4.51, p<0.05). Aging promoted depressive-like behaviours as indicated by less time spent climbing (Figure 1D, 1E, 1F; young vs. middle-aged, p<0.01; middle-aged vs. aged, p<0.001). Males generally spent more time climbing than females but MPS significantly reduced climb time in aged males (p<0.01; Figure 1E, 1F). Thus, MPS had the most impact on emotional state after midlife in males, while MPS females mainly showed resilience.

### MPS exacerbated age-associated impairments in skilled walking in males

MPS and aging altered both forelimb (FL) and hindlimb (HL) placement in the ladder rung walking task (Figure 1G) with sex-specific outcomes. A mixed longitudinal assessment at 6 (young), 12 (middle-aged) and 18 months (aged) of age in the ladder rung task demonstrated significantly lower FL ladder rung placement scores in middle-aged and HL ladder rung placement score in aged MPS male rats, but not in females. A three way-ANOVA showed a main effect of SEX, as females had more accurate FL (F(2,125)=27.5, p<0.0001) and HL (F(2,125)=24.3, p<0.001) motor control than males. A main effect of AGE was found for FL (F(2,125)=10.4, p<0.001) and HL (F(2,125)=5.31, p<0.01) placement; younger rats displayed the highest FL and HL placement scores, which diminished with age in males (Figure 1H, 1I). FL placement scores diminished in MPS groups (F(2,125)=3.69, p<0.05). MPS males had higher FL placement scores at 6 (p<0.05) and lower at 12 months of age (p<0.05) compared to CONTROL. HL placement was impaired in males at all ages, but most severely at old age (p<0.05). These findings show that aging and MPS synergistically impaired coordinated locomotor abilities and balance in males only.

### MPS and aging synergistically altered gross motor activity

Open field assessment in exploratory behaviour at 6 (young), 12 (middle-aged) and 18 months (aged) of age showed synergistic age and MPS effects on gross motor function. A three way-ANOVA showed main effects of AGE (F(2,102)=54, p=0.001), SEX (F(2,102)=132, p=0.000), STRESS (F(2,102)=6.43, p=0.013) and AGE x SEX interaction (F(2,102)=7.7, p=0.001) in open field exploratory activity. Aging reduced activity across all groups; aged rats travelled shorter distances than young (p<0.001) or middle-aged (p<0.05) rats. Overall, females were twice as active as males. MPS animals across all ages travelled longer distances than age-matched CONTROLs (Figure 1J). Thus, MPS induced distinct sexually dimorphic exploratory profiles with highest sensitivity in females.

### Physiological health outcomes

### MPS regulated the stress response as a function of sex and age

**Figure 2:**
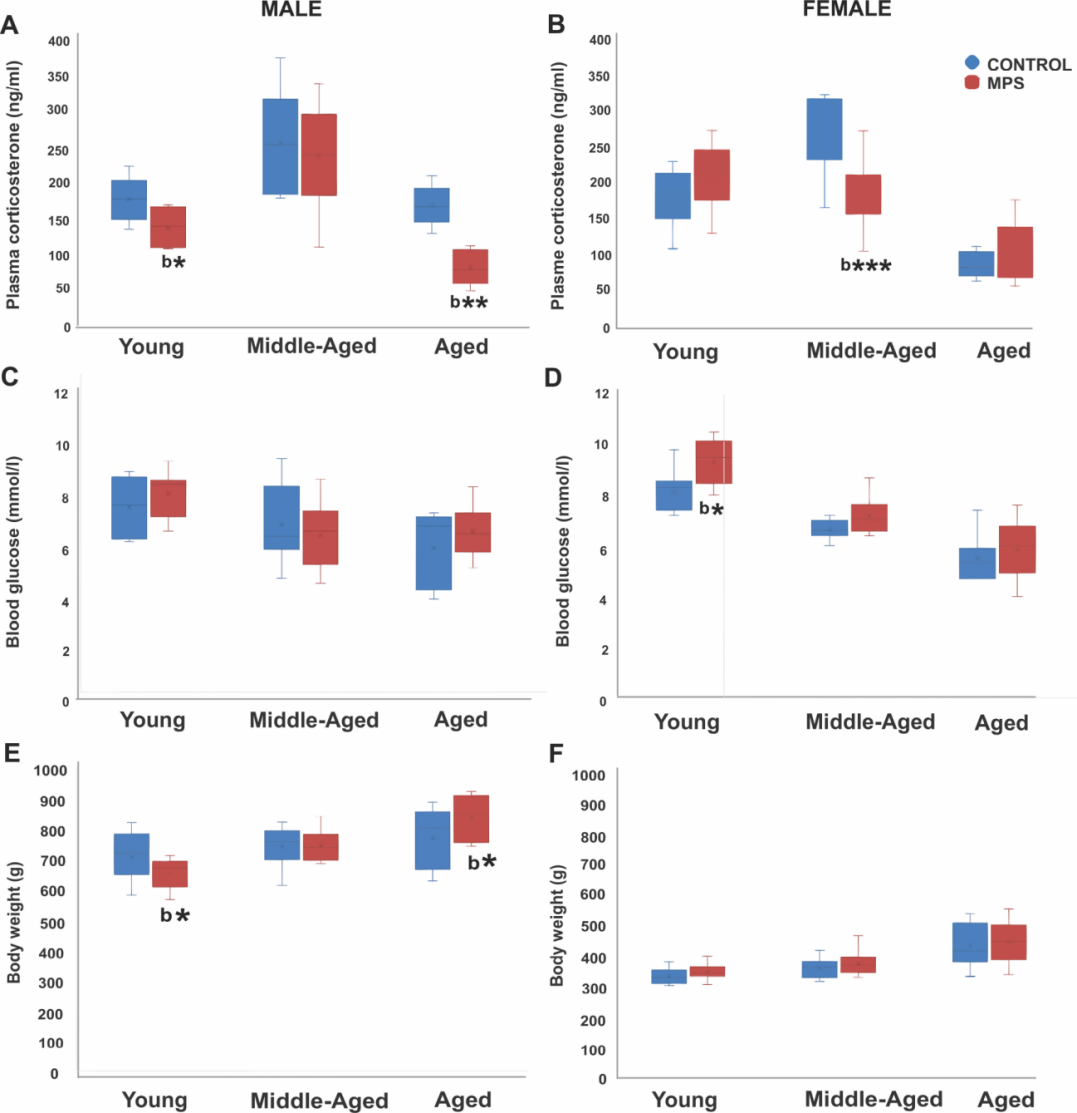
**MPS determines physiological health trajectories.** Plasma corticosterone and blood glucose levels and body weight in male and female rats revealed an effect of MPS across the lifespan. (**A**, **B**) MPS induced sex- and age-specific modifications in the stress response as indicated by reduced plasma corticosterone levels in young and aged males (**A**), and middle-aged females (**B**). (**C**, **D**) MPS elevated non-fasting blood glucose levels especially in young females. (**E**, **F**) MPS diminished body weight in young and increased it in old males (**E**). Body weight in males and females increased with age, while males weighed twice as much as females. Asterisks indicate significances: *p<0.05, **p<0.01, ***p<0.001; “b” indicates MPS effect.

Plasma corticosterone assessment at 6 (young), 12 (middle-aged) and 18 months (aged) of age revealed age- and sex-specific MPS effects. A three way-ANOVA showed a main effect of AGE (F(2,104)=50.3, p<0.001) and STRESS (F(2,104)=7.18, p<0.01), but no effect of SEX. Middle-aged animals showed the highest and aged animals the lowest values, while MPS generally reduced basal corticosterone levels compared to CONTROLs. An interaction between AGE x SEX (F(2,104)=4.21, p<0.05), SEX x STRESS (F(2,104)=4.5, p<0.05) and AGE x SEX x STRESS (F(2,104)=6.7, p<0.05) indicates that MPS affected males and females differently depending on age. MPS reduced circulating corticosterone levels in young and aged male rats (p<0.01; Figure 2A) while MPS females showed reduced midlife corticosterone levels (p<0.01; Figure 2B). These results indicate that MPS overall blunted the stress response in animals with an ancestral history of stress. The stress response of MPS males was most sensitive at young and old age, while MPS females were most affected in midlife.

### MPS reversed age-associated effects on circulating blood glucose levels in females

Assessment of circulating blood glucose levels at 6 (young), 12 (middle-aged) and 18 months (aged) of age revealed that an age-associated decrease in glucose levels was reversed by MPS in females. A three way-ANOVA showed main effects of AGE (F(2,108)=44.9, p<0.001), STRESS (F(2,108)=6.13, p<0.05), and SEX x AGE interaction (F(2,108)=4.87, p<0.01). Aging decreased blood glucose levels; young animals had higher glucose levels than middle-aged and aged animals (p<0.001; Figure 2C, 2D). Notably, MPS females had higher glucose levels at all ages than CONTROL females and highest values in the young (p<0.01; Figure 2C, 2D), while no effects were observed in males. These results suggest that a history of ancestral stress may represent a potential risk factor for diabetes.

### MPS showed sex-and age-specific growth in body weight

Aging was generally associated with an increase in body weight at 6 (young), 12 (middle-aged) and 18 months (aged) of age. MPS changed body weight gain in males only, with making them slimmer early in life, and heavier in old age compared to CONTROLs. A mixed three way-ANOVA revealed main effects of AGE (F(2,135)=38.9, p<0.001) and SEX (F(2,135)=12.94, p<0.001), but no effect of STRESS. Aging increased average body weight by about 50 g (p<0.001; Figure 2E, 2F). Notably, MPS males had lower body weight at 6 (p<0.05) and 12 months of age (p>0.05; Figure 2E) than CONTROL males. However, at 18 months of age, MPS males experienced substantial body weight gain (p<0.05) and surpassed the body weight of CONTROLs. These results suggest that MPS males may be programmed to be more fit and healthier earlier in life at a cost of old age obesity.

### Mortality and disease incidence

### MPS generated sex-specific midlife mortality and lifetime survival probability

**Figure 3:**
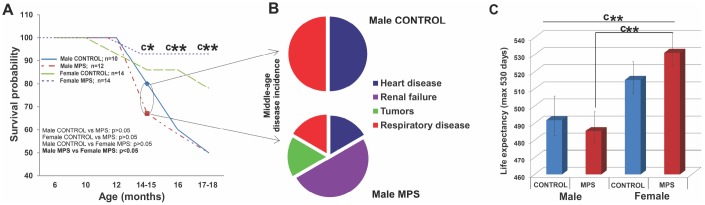
**MPS determines sex-specific morbidity and mortality.** (**A**) MPS males were more likely to die prematurely than any other group (at 14-15 months). (**B**) Midlife premature death in MPS males was linked to higher disease risk for renal failure, heart and respiratory disease and tumors. (**C**) Life expectancy with 530 days maximum endpoint. Asterisks indicate significances: *p<0.05, **p<0.01, ***p<0.001, “c” indicates survival probability of MPS males vs. MPS females age 14-15 (*), 16 (**) and 17-18 months (**). Male CONTROL n=10, male MPS n=12, female CONTROL n=14, female MPS n=14.

Health status and outcomes including morbidity and mortality were monitored across the lifespan (0-18 months of age) and incidental mortality (before 18-month experimental endpoint) was recorded. MPS animals showed sex-specific survival probability in midlife. MPS males were 33% more likely to die by the age of 14 months, compared to 7% of MPS females (Figure 3A). Premature mortality in middle-aged MPS males was linked to multiple pathologies, including renal failure (2/12) (Figure 3B), heart disease (1/12), respiratory disease (1/12) or tumors (1/12). By contrast, in CONTROL males (n=10) premature death was induced by sudden heart attack (1/10) or respiratory disease (1/10; Figure 3B). In females (n=28) all premature deaths were due to renal failure, affecting one MPS (1/14) and two CONTROL females (2/14).

Kaplan Meier and Cox regression tests (p<0.05; HR=0.33) revealed that the lifetime probability of death was 67% lower in females than males. MPS males died more likely at middle age (33%; n=4/12) than CONTROL males (20%; n=2/10). At 14 months, only 67% (n=8/12) of MPS males and 80% (n=8/10; Figure 3A) of CONTROL males were still alive. At 17-18 months only 50% of MPS and 50% of CONTROL males were still alive (Figure 3A). MPS had only slight effects on female mortality at middle age (alive CONTROL, n=12/14; MPS, n=13/14) and somewhat positive effects at 18 months (alive CONTROL, n=10/14; MPS, n=13/14).

Lifespan data showed a main effect of SEX for the total number of days alive (F(1,46)=5.7, p<0.05 (Figure 3A, 3C) as females overall had a longer lifespan than males. MPS males and MPS females were significantly different (t(26)=-2.8, p<0.01; Figure 3C). At the experimental endpoint of 530 days, MPS increased mortality in males with only 5/10 males surviving, compared to 13/14 females. Thus, MPS but not sex accounted for the shorter lifespan observed in males.

### MPS raised the risk of inflammatory, renal and respiratory disease across the lifespan

**Figure 4:**
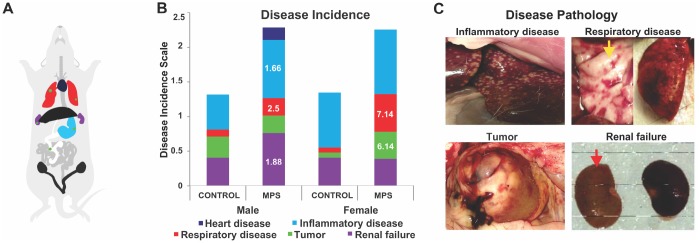
**MPS alters disease incidence across the lifespan.** (**A**) Diagram illustrating the colour code of organ pathophysiological changes. (**B**) Disease incidences as represented by respective colours. The relative risk (RR) in white letters indicates diseases prevalence in relation to CONTROLs. (**C**) Photographs of disease pathology in MPS animals, illustrating enlarged spleen, alveoli changes in lung disease (yellow arrow), abdominal tumor, and kidneys linked to renal failure (red arrow).

Disease incidence and disease pathologies were recorded after the institutional veterinarian confirmed diagnosis of post-mortem organs and tissues. Relative risk analyses of disease incidence demonstrated increased risk for inflammatory, renal and respiratory disease (Figure 4A) in MPS males and a higher risk of respiratory disease and tumours in MPS females. MPS males were 1.66 times more likely to suffer from inflammatory disease (CI: 0.85-3.25; Figure 4B, 4C) and 1.88 times more likely to have renal failure (CI: 0.82-4.28; Figure 4B) than CONTROL animals. The risk of respiratory disease was 2.5 higher in MPS than CONTROL males (CI: 0.305-20.4; Figure 4B). The risk of respiratory illness and tumors were 7.14 (CI: 1.10-49.6; Figure 4B, 4C), and 6.14 (RR=6.14; CI: 0.825-43.5; Figure 4B, 4C) times higher among MPS females than CONTROL females; respectively. Thus, MPS increased the sex-specific risk of disease across the lifespan.

### Up-stream epigenetic regulation

### MPS induced sex- and age-specific epigenetic programming by miRNA

**Figure 5:**
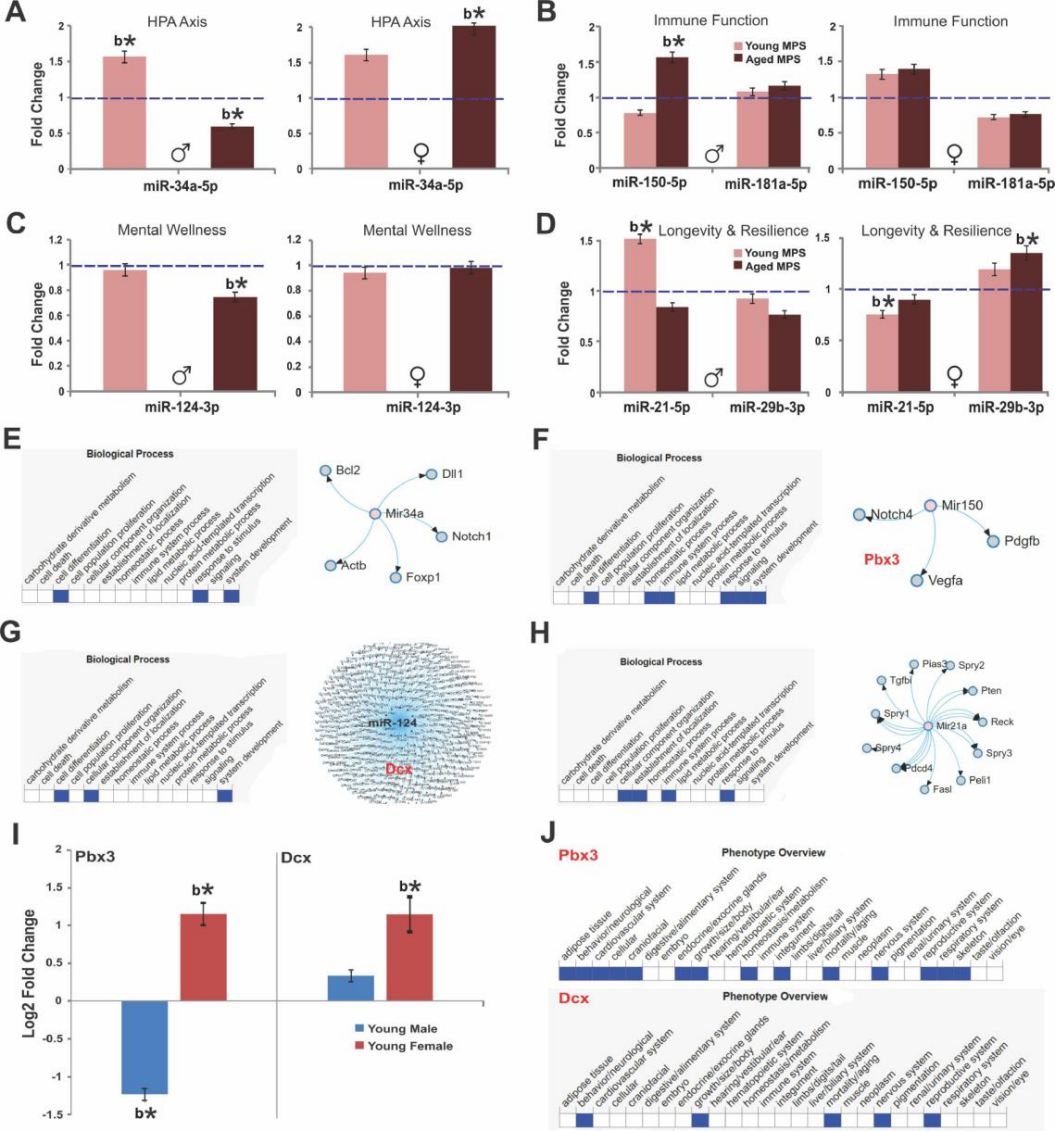
**MPS raises age-associated stress vulnerability via sex-specific miRNA and mRNA expression.** Fold change of miRNA and mRNA expression as determined by deep sequencing of prefrontal cortex. (**A**) Expression of miR-34a, (**B**) miR-150 and miR-181a, (**C**) miR-124, (**D**) miR-29b indicate MPS-programming of aging trajectories. Biological processes and miRNA targets are shown for (**E**) miR-34a, (**F**) miR-150, (**G**) miR-124, and (**H**) miR-21. (**I**) Sex-specific changes in the expression of pre-B-cell leukemia transcription factor 3 (*pbx3)* and *doublecortin (dcx)* genes as a function of age. (**J**) Phenotype overview of *pbx3* and dcx as per MDB database. All data are represented as log change relative to CONTROL levels. Blue, dashed line indicates age-specific CONTROL levels. Asterisks indicate significances: *p<0.05, **p<0.01. All data are presented as mean ± SEM, “b” indicates MPS effect, n=3 per age/sex/group.

From a subset of male and female young (6 months, n=12) and aged (18 months, n=12) animals (3 per group) frontal cortex tissue was collected for epigenetic profiling (miRNA and mRNA). Whole tissue deep sequencing revealed that nine cortical miRNAs were differentially regulated by MPS: 1) miR-150-5p, miR-181a-5p and miR-181c-5p that influence immune function through B and T cell regulation [46, 47]; 2) miR-34a-5p, miR-34c-5p, and miR-124-3p involved in HPA axis response, synaptic plasticity and mental health [26, 48–50]; 3) miR-29a-3p and miR-29b-3p that regulate DNA methylation and resilience [51]; 4) the senescence/longevity biomarker miR-21-5p ([52]; Figure 5).

MPS animals (males, young and aged; females, young and aged) were compared to CONTROLs using the Benjamin and Hochberg corrections for p-value adjustments. In young males, MPS upregulated miR-34a-5p (false discovery rate adjusted [FDR] p<0.05; Figure 5A) and miR-21-5p (FDR p<0.05; Figure 5D). In young females, MPS downregulated miR-21-5p (FDR p<0.05; Figure 5D) and miR-181a-5p (FDR p>0.05 Figure 5B), while miR-29b-3b, miR-34a-5p and miR-150-5p (FDR p>0.05; Figure 5) were upregulated in comparison to CONTROL levels.

More profound alterations were observed at old age. MPS in males downregulated miR-34a-5p (FDR p<0.05; Figure 5A) and miR-124-3p (FDR p<0.05; Figure 5C) and upregulated miR-150-5p (FRD p<0.05; Figure 5B). In females, MPS upregulated miR-29b-3p and miR-34a-5p (FDR p<0.05; Figure 5) and slightly downregulated miR-21-5p and miR-181a-5p (p>0.05; Figure 5B, 5D). Figure 5E summarizes the putative target genes of miR-34a emphasizing its role in growth factor signaling and brain development through *bcl2* and *notch1* regulation [48]. MPS upregulated expression of miR-150 and downregulated its target gene pre-B cell leukemia homeobox 3 ([53, 54]; *pbx3*; Figure 5F, 5I) in young males (FDR p<0.05) and downregulated pbx3 in young females (FDR p<0.05). MPS upregulated miR-124 targeting the doublecortin (*dcx*) gene and various others in young males and females (FDR p<0.05; Figure 5G, 5I). Finally, the senescence/longevity marker miR-21a interacts with cell signaling and immunity pathways ([52, 55, 56]; Figure 5H). These changes link MPS experiences to brain development and resilience as *dcx* regulates microtubule-based vesicle transport (Figure 5J), a process critical to both neuronal migration and axonal outgrowth [57]. These data show that MPS via homeostatic regulation accelerates aging and senescence in males and enables lifetime resilience in females.

### Down-stream cellular metabolomics

### MPS altered metabolic pathways linked to disease risk at young age

**Figure 6:**
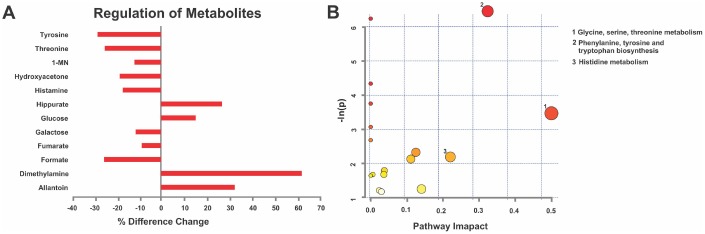
**MPS defines cellular homeodynamics as reflected by deep learning metabolomics analysis.** (**A**) Given their disease vulnerability, males revealed a characteristic metabolic signature in urine ^1^H-NMR spectra based on VIAVC testing. Individual metabolite changes are indicated; bars indicate % change from CONTROLS. (**B**) Pathway topology analysis showing all matched pathways according to p-values and pathway impact values in young males. This figure was created using the lists of metabolites identified in A. n=7 CONTROL and n=6 MPS rats; 1-MN 1-methylnicotinamide.

For early prediction and prevention, it is important to identify cellular pathogenic pathways prior to disease onset. Thus, post-mortem urine was collected via bladder puncture from young (6 months) male and female rats. Following ^1^H NMR spectroscopy [24] the data were exported to undergo Variable Importance Analysis based on random Variable Combination (VIAVC) for metabolite identification and KEGG pathways analysis. MPS in young males resulted in unique metabolic profiles when compared with CONTROLs, including 12 metabolites associated with immune regulation and senescence, such as dimethylamine, hippurate, histamine and threonine (p<0.05; Figure 6A). MPS reduced eight and elevated four metabolites (Figure 6A). Pathway topology analysis found multiple metabolic pathways predicted health trajectories in a genome-scale network model of rat metabolism (Table 1). MPS in young males modified phenylalanine, tyrosine and tryptophan biosynthesis (p<0.05), glycine, serine and threonine (p<0.001) and histidine (p>0.05; Figure 6B) metabolisms. Thus, MPS altered metabolic pathways potentially linked to disease risk at young age.

**Table 1:** Metabolic pathways altered by MPS in young males.

**Pathway Name**	**Total**	**Hits**	**p-Value**	**Impact**
Glycine, serine and threonine metabolism	32	3	0.0015756	0.3236
Methane metabolism	9	2	0.0019564	0
Aminoacyl-tRNA biosynthesis	67	3	0.013068	0
Ubiquinone and other terpenoid-quinone biosynthesis	3	1	0.02337	0
Phenylalanine, tyrosine and tryptophan biosynthesis	4	1	0.031049	0.5
Cyanoamino acid metabolism	6	1	0.046243	0
Phenylalanine metabolism	9	1	0.068627	0
Histidine metabolism	15	1	0.11197	0.22043

“Total” indicates the number of metabolites listed in the pathways. “Hits” indicate the number of significant metabolites identified in the pathways. The p-value is based on metabolite enrichment analysis. “Impact” designates the pathway impact as determined by pathway topology analysis.

## DISCUSSION

Through epigenetic programming, environment and lifestyle can become major determinants of lifelong health. The present findings for the first time confirm and support the notion that mutigenerational ancestral adverse experiences accelerate age-associated mental and physical health decline. Aging *per se* increased the risk of anxiety- and depressive-like behaviours and reduced locomotor capacities, limb coordination and balance. These deficits were further exacerbated by MPS reaching back four generations (F0-F3), particularly among males, and coincided with epigenomic, transcriptomic and metabolomic signatures of senescence, stress vulnerability, immune function and emotional regulation [58]. Thus, epigenetic programming by ancestral stress, including miRNA modifications, is arguably a determinant of sex-specific lifetime health trajectories and risk of common age-related NCDs.

The present results show that aging promoted changes in emotional phenotype, as indicated by excessive margin time in the open field and helplessness in the forced swim task in males, and increased helplessness in middle-aged female rats. These changes were exacerbated by exposure to MPS in a sex-specific manner, as young and aged MPS males showed the highest levels of anxiety-like behaviour and aged MPS males showed increased depressive-like behaviours with no effects in females. These findings resemble clinical observations made in offspring exposed to prenatal stress, undernutrition, infection or environmental chemicals, all of whom show increased risk of anxiety and depression [22, 28, 59–61]. In addition, males were more vulnerable to stress than females [62–64] in association with potentially predictive metabolic signatures linked to brain function (e.g., changes histidine) [65]. Unbiased deep sequencing revealed core epigenetic regulatory pathways altered by ancestral stress linked to behavioural and physical phenotypes. MiR-21 and miR-34a stood out as regulators of the consequences of MPS. MiR-21 represents a marker of longevity via regulation of down-stream biomarkers of senescence and aging [66]. Clinical studies demonstrate individuals who age successfully to 80+ years display down-regulated miR-21 [50, 66]. Thus, the present miR-21 down-regulation in females may provide a mechanistic link to lower mortality, which by regulating the p53 pathway, may prevent tumorigenesis and maintain genomic integrity [66, 67]. In contrast, upregulated miR-21 in males may result in premature aging and associated pathologic conditions [66, 67]. Accordingly, antagomirs directed against miR-21 prevent pulmonary disease, cardiac fibrosis and renal fibrosis [55, 68].

Although miRNAs were the focus of this study, other epigenetic mechanisms including histone modification and DNA methylation may have been involved in generating the observed phenotype, and DNA methylation and miRNA expressions can reciprocally influence each other [69–72]. Transgenerational mechanisms that determine successful aging arguably include stress-induced DNA methylation, involving DNA methyltransferase 3 alpha (DNM3a) or DNA methyltransferase 3 beta (DNMT3b) of primordial cells and sperm to provide heritable markers [73]. Although the present study did not examine germ cell DNA methylation, the sex-specific changes in cortical miR-29 expression known to modulate DNMT3a and DNMT3b expression may underline ancestral inheritance [74, 75]. Importantly, miR-29 regulates DNMT3 activity in primordial cells of females rather than males [76]. Here, MPS promoted upregulation of miR-29 expression in females but downregulated in males, which may indicate programming by stress in early development particularly in female lineages. MiR-29 alterations regulate somatic mutations of DNMT3a, associated with pathological states including immunodeficiency syndrome [51, 77, 78], and manipulating miR-29 expression may have therapeutic applications [67]. It remains to be tested if these differences may have protected female offspring against adverse health outcomes.

The present study also found miR-34a, as a marker of the emotional stress response, upregulated in young males and females but downregulated in aged males. miR-34a is implicated in anti-apoptotic actions, with enrichment particularly in aged cells [48]. miR-34a may also facilitate adaptation to environmental stressors by its involvement in stimulus response processes [79] and regulate the expression of corticotropin-releasing hormone receptor-1 gene (*crhr1)* [50, 80, 81]. Its upregulation in young MPS males and females may indicate reduced CRH expression contributing to higher anxiety-like behaviour [50], a consequence of reprogrammed endocrine system activity by ancestral experience. Here, miRNAs represent not only sex- but also age-specific biomarkers. Upregulated miR-150 serves as the most age-sensitive immune marker in MPS males. In females, this marker was upregulated by MPS independent of age. At any age, MPS downregulated miR-181. Both miR-150 and miR-181 are involved in fine-tuning adaptive immune response, with miR-150 being mostly expressed in mature B and T cells [67, 74–82] suggesting that stress-related miR-150 overexpression may promote risk of autoimmune dysfunction, resulting in premature morbidity and mortality. Specially, miR-150 targets gene pre-B-cell leukemia transcription factor 3 (*pbx3*), further regulating immune response, homeostasis and metabolism including incidence of diabetes and mortality from cancer [83, 84].

The combined miRNA and mRNA profiles in MPS males suggest altered inflammatory activity, or more specifically induced a pro-inflammatory state [1, 4, 5, 85, 86]. These observations are supported by stress-related incidence of inflammatory disease in males, but not females, over the life course. The observed heightened risk of inflammatory disease in MPS males was similar to the average risk of morbidity in females independently of stress. At the cellular level, reduction of histamine and threonine may be linked to higher demand by the immune system and increased susceptibility to disease [87]. MPS increased hippurate expression as previously reported in diabetes and kidney failure [88]. At 14 months of age, 33% of MPS males had died compared to 20% of non-stressed males, while 95% of females lived to the end of the experiment at 18 months of age. Similarly, exposure to the environmental endocrine disruptor vinclozolin promoted mammillary and prostate tumours, and kidney and immune disorders in 6-12 month old F1-F4 rat offspring [73, 89]. The interactive nature between the immune system and androgens [90, 91] may explain sex-specific disease incidence. In turn, as estrogen modulates T cell activity, and promotes T-helper 2 differentiation [90], females become more prone to autoimmune disease [91]. The present study found a higher incidence of respiratory disease and tumors in MPS females, suggesting both conditions may be associated with upregulated *pbx3* gene expression [83, 84]. A causal unifying mechanism for the complex experience-dependent phenotype in MPS-treated animals arguably involves maladaptive HPA axis programming. The reduced corticosterone levels at advanced ages indicate chonic wear-and-tear of the HPA axis in MPS similar to post-traumatic stress disorder (PTSD; [92]. Hence, MPS in males may blunt basal HPA axis activity thus compromising adaptive stress response at particularly vulnerable times in life [93]. Accordingly, we demonstrated previously that MPS upregulates cortical *chr* gene expression in adult males, but not in females [26]. These findings suggest a characteristic age-dependent profile of stress markers to be considered in the prediction and diagnosis of stress-related NCDs.

Down-stream consequences of ancestral stress on aging and disease may be linked to sex-specific metabolic homeodynamics [34, 94, 95] via altered glucose levels [96]. Dysregulated blood glucose levels associated with higher risk of diabetes have been observed following early life adversity in clinical and experimental studies [14, 97, 98]. F4 trans- and multigenerational stress adult males displayed altered histamine and hippurate levels [24] which are involved in glucose and insulin regulation [99, 100]. Moreover, MPS exaggerated aging effects on body weight [101, 102], as stressed females were slightly heavier at each age than non-stressed counterparts. Stressed males, however, had lower body weight at young ages and then grew significantly heavier with age. Abdominal obesity, diabetes and heart disease may result from stress-associated changes in feeding behaviour and caloric intake [103].

Therefore, the present study demonstrates that ancestral stress is an important determinant of lifetime health trajectories. Young and old age are particularly vulnerable periods in life to display a stress-related phenotype, with accrued homeodynamic challenges promoting morbidity and mortality especially in later life. MPS accelerated aging processes in males and partially spared females. Mechanisms of stress vulnerability in males and potential resilience in females include altered HPA axis response and altered inflammatory status. MPS in males resembled phenotype features of human PTSD proposing its value as a new animal model for this condition. miRNAs via regulation of mRNA and metabolomic expression may play a key role programming stress vulnerability through the maternal lineage and provide new predictive biomarkers of age-related NCDs suitable for consideration in precision medicine approaches. The present data emphasize that aging and NCD risk are not only influenced by genetics and lifestyle but also by experiences in previous generations.

## MATERIALS AND METHODS

### Experimental design

This mixed longitudinal study involved 88 Long-Evans hooded rats (42 males, 46 females). F4 generation male and female offspring were derived from two lineages bred under standardized conditions: multigenerational prenatal stress (MPS) and non-stress yoked CONTROL. Prenatal stress was induced by subjecting pregnant dams to semi-random daily 5 min swim and 20 min restraint stress in a Plexiglas cylinder from gestational days 12-18 [3, 17, 24, 26, 93].

**Figure 7:**
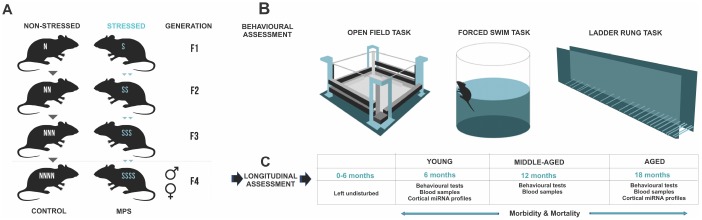
**MPS rat lineages and experimental design**. (**A**) The MPS lineage was generated by stressing pregnant dams over four consecutive generations (F0, F1, F2, F3) to produce multigenerationally stressed (MPS) F4 offspring. Yoked non-stress CONTROL F4 offspring were generated in parallel. (**B**) Behavioural phenotype was assessed in open field exploratory behaviour, forced swim task learned helplessness, and ladder rung skilled walking. (**C**) MPS and CONTROL F4 generations were used for a mixed longitudinal experiment with tests at 6, 12 and 18 months of age.

Five generations of timed-pregnant female rats were bred under standardized conditions to generate the multigenerational prenatal stress (MPS) F4 offspring (Figure 7A). Parental female rats (F0) were stressed during late gestation (GD 12-18) to generate F1-S female daughters (Figure 7A). Pregnant F1-S female daughters were stressed during late gestation to generate F2-SS female granddaughters. Pregnant F2-SS female granddaughters were stressed during late gestation to generate F3-SSS great granddaughters. Lastly, F3-SSS great daughters were stressed during late gestation to generate the F4-SSSS or F4 multigenerational prenatal stress (MPS) male and female great-great grand offspring. A lineage of yoked controls was bred parallel to each generation (non-stress pregnant F0, F1-N, F2-NN, F3-NNN) to generate F4-NNNN or CONTROL male and female offspring. Each generation F0-F4 was outcrossed avoiding inbreeding by at least four generations. Distinct lineages were monitored through the JAX Colony Management System (JCMS; Jackson Laboratory, Bar Harbour, ME, USA). A maximum of three offspring per litter of each sex were randomly selected to be included in the experiments. Each experimental group included offspring from at least 3-4 different litters. Bystander effects of stress were avoided by using designated testing and housing spaces. All housing, handling, testing and tissue sampling conditions were harmonized across generations.

These F4 generation offspring were tested at 6 (young), 12 (middle-aged) and 18 (aged) months of age (Figure 7). One subgroup of F4 offspring (young [males: n=20 (CONTROL=10, MPS=10); females: n=18: (CONTROL=10, MPS=9] underwent behavioural testing in open field, forced swim task and ladder rung walking task (Figure 7B) and blood collection at 6 months of age. They were then euthanized for fresh tissue and urine collections. A second subgroup of F4 offspring was left undisturbed with only weekly weighing and cage changes up to 12 months or middle-aged [males: n=22 (CONTROL=10, MPS=12); females: n=28 (CONTROL=14, MPS=14)]. At 12 months and 18 months of age these F4 offspring underwent behavioural assessments in open field, forced swim task and ladder rung walking task and blood collection (Figure 7B). Glucose levels and plasma corticosterone levels were determined at each time point and animals were weighed weekly.

In this mixed-longitudinal study, the behavioural and physiological data were analyzed by mixed three-way ANOVA. Group sizes for all *in vivo* tests were: (1) young [males: n=20 (CONTROL=10, MPS=10); females: n=18: (CONTROL=10, MPS=9], (2) middle-aged [males: n=22 (CONTROL=10, MPS=12); females: n=28 (CONTROL =14, MPS=14)] and (3) aged [males: n=13 (CONTROL =6, MPS=7); females: n=18 (CONTROL=10, MPS=8)]. A subset of animals was randomly assigned for epigenetic analyses [male n=12 (CONTROL=6, MPS=6); female n=12 (CONTROL=6, MPS=6)] at 6 and 18 months of age (n=3 per time point).

All tests were performed by an experimenter blind to the experimental conditions. Overall health status was inspected by the institutional veterinarian and recorded. Disease incidence and pathologies were recorded after the institutional veterinarian confirmed diagnosis of post-mortem organs and tissues. All procedures were approved by the University of Lethbridge Animal Care Committee in compliance with the guidelines by the Canadian Council on Animal Care.

### Behavioural testing

### Exploratory activity and anxiety-like behaviour

Exploratory activity and anxiety-like behaviours were assessed in an open field task, a standard measure of emotional state and overall locomotor activity in rats (Figure 1A; [16]). Animals were placed in Accuscan activity monitor Plexiglas boxes (length 42 cm, width 42cm, height 30 cm) and their activity was recorded for 10 min using VersaDatTM software (AccuScan Instruments Inc., OH, USA). Horizontal distance travelled and time spent in margins were recorded and used for quantification of overall explorative activity and anxiety-like behaviour respectively. The average distance traveled (cm) and average time (sec) spend in the margin zone were used in the analysis and represented in Figure 1B, 1C and 1J.

### Depressive-like behaviour

Self-helplessness and depressive-like behaviours were recorded using the Porsolt swim task (forced swim task, FST; Figure 1D; [104]). Animals were placed in a cylinder containing warm water (21°C) and videorecorded for 5 min. Video recordings were analyzed for the time (sec) spent climbing the walls of the cylinder. The results are represented in Figure 1E and 1F.

### Skilled walking

Qualitative skilled fore- and hindlimb placements were assessed using the ladder rung walking task (Figure 1G; [3, 105]). Animals were pre-trained and the next day tested three times at each age time point. Video recordings were analyzed for qualitative placement scores [left forelimb (LFL); right forelimb (RFL); left hindlimb (LHL); and right hindlimb (RHL)] by an observer blind to the experiment. The statistical analysis was initially run on each forelimb (LFL, RFL) and hindlimb (LHL and RHL) and since no statistical significance was observed between left and right limbs, the two were averaged. The data presented in Figure 1H and 1J show average forelimb and hindlimb placement scores.

### Blood analysis

Blood samples were obtained three days prior to behavioural testing at each age (Figure 7C). Approximately 0.6 ml of blood was collected from the tail vein in the morning hours between 8:00 and 10:00 AM under 4% isoflurane anaesthesia [17, 106]. Blood glucose was measured using an Ascensia Breeze Blood Glucose Meter with test strips (Bayer, ON, Canada). From the remaining blood, plasma was obtained by centrifugation at 5,000 rpm for 10 min. The samples were stored in -80 °C. Plasma was isolated and corticosterone levels were determined by enzyme-linked immunosorbent assays (ELISA; Cayman Chemical, MI, USA).

### Organ collection

Animals were euthanized with an overdose of pentobarbital (Euthansol 100 mg/kg; CDMV Inc., Québec, Canada). Brains, kidney, liver and lungs were rapidly removed, dissected and flash frozen. Remaining organs and tissues were saved for post-mortem diagnoses of pathologies by the institutional veterinarian, who was blind to treatment groups.

### mRNA and miRNA deep sequencing

Following behavioural testing, one subgroup of animals [(young, n=3 per treatment group/sex; aged, n=3 per treatment group/sex) was treated with an overdose of pentobarbital (Euthansol 100 mg/kg; CDMV Inc., Quebec, Canada), and once the vital signs were discontinued animals were decapitated. The brains were rapidly removed, dissected, and flash-frozen for miRNA and mRNA analysis. TRI Reagent Solution (Invitrogen, Carlsbad, CA, USA) was used to extract total RNA from frontal cortices. mRNA and miRNA expression was analysed by an Illumina GAIIx genomic analyzer (Illumina 462 Inc., San Diego, CA, USA). Briefly, for miRNA, base calling and demultiplexing was completed using CASAVA 1.8.1 software (Illumina 462 Inc., San Diego, CA, USA). FastQC software was used to examine short read quality. Adapters were trimmed using Cutadapt software [107]. miRNA first raw counts underwent normalization and variance stabilization as per DESeq2 [108]. Another FastQC quality check was preformed after trimming. MicroRazerS version 1.0 [108] was used to perform miRNA mapping. Reads mapping to mature miRNA were counted using an ad hoc bash script. Potential targets of selected miRNAs of interest were predicted using the 3 ‘UTR available for Rat rn5 (UCSC) genome. An algorithm (miRanda v.3.3a, Computational Biology Centre of Memorial Sloan-Kettering Cancer Centre, NY, USA) was used for miRNA target prediction. Mouse Genome Database [58] was used to create biological process, phenotype overview and miRNA target diagrams.

For mRNA, every library was sequenced across 3 lines using multiplex. Base calling and demultiplexing was performed by Illumina CASAVA 1.8.1 with default settings using Rat-Rnor 5.0 (Ensembl) and reference, sequence and annotation information were downloaded from iGENOME (Illumina 462 Inc., San Diego, CA, USA). Raw count data were uploaded into R, initial data exploration and outlier detection were preformed using arrayQualityMetrics and DESeq2 Bioconductor package (http://www.bioconductor.org). First raw counts underwent normalization and variance stabilization procedure as described in DESeq2 manual. Hierarchical clustering of transcriptional profiles based on top 100 most variable genes, pre-selected from the subset of highly expressed genes (higher than a median expression). Clustering was preformed using heatmap.2 function from gplots package (RDocumentation) with default clustering algorithms. In addition to hierarchical clustering, similarities between samples were visualized as principal component analysis (PCA) plots using plot PCA function implemented in DESeq2. Outlier detection and transcriptional profile quality control was preformed using array Quality Metrics package (Bioconductor).

### Urine NMR spectroscopy

Urine samples were obtained through bladder puncture with a 1.5-ml sterile syringe at time of euthanization and stored at −80°C. Samples were prepared for NMR spectroscopy [24] on a 700 MHz Bruker Advance III HD spectrometer (ON, Canada) and the data exported to MATLAB (MathWorks, MA, USA). Variable Importance Analysis based on random Variable Combination (VIAVC) was used as a feature/bin selection method [109]. Metabolite identification used Chenomx 8.2 NMR Suite (Lethbridge, AB, Canada) followed by Metaboanalyst [110] and Kyoto Encyclopedia of Genes and Genomes (KEGG) pathway library for rats.

### Statistical analysis

Three-way mixed ANOVA with sex, stress, and age as factors was run for behavioural tasks, corticosterone and glucose levels, and body weight. Tukey’s post hoc test and independent sample t-test were used for all behavioural and physiological post-hoc analyses. Survival probability was assessed using Kaplan-Meier survival curves and Cox Regression was performed to calculate the hazard ratios (HR). Survival rate at 14-15 months of age was assessed using Fisher’s exact test. Relative risk (RR) and confidence intervals (CI) were calculated for each disease. For miRNA and mRNA analysis, raw count data was first normalized and regularized with log transformation using statistical routines implemented in the DESeq2 Bioconductor package [108]. Default settings were used to perform normalization and statistical analysis. Pairwise comparison between experimental group (MPS vs. CONTROL) were performed using DESeq2. To be considered differently expressed, miRNA and mRNA with a false discovery rate adjusted p-values <0.05 were used. All results are shown as the means ±standard error of the mean (±SEM).
